# Case of Enlarged Liver

**Published:** 1816-12

**Authors:** John Bailey

**Affiliations:** Surgeon


### *CASE of ENLARGED LIVER.*

By John Bailey, Surgeon.

A Fine and remarkably healthy-looking little boy, aged six years, son of a gentleman of this town, became triflingly indisposed in the Christmas holidays of 1814. A dose or two of mercurial physic gave him relief, and he appeared to be well again. A fortnight afterwards, he became chilly and languid, and disinclined to pursue those objects with which boys are generally amused: he did not complain of any pain; his pulse grew quick; there was some fever, and the tunicæ conjunctivæ were suffused with yellowness. At this period he took saline medicine, combined with small doses of hyd. subm. and occasional aperients, to overcome a costive state of the bowels; in three or four days the salivary organs became seriously affected, so that it was necessary to lay aside the mercurial course. As the operation of this medicine diminished upon the system, the child grew better, the yellow appearance in the eyes vanished, and his general health was so much improved, that although not in *perfect health,* he was sent to school, in the hope that a change of scene and of air, and the sight of former companions, might conduce to hasten the return of sound health. During his continuance there until the summer holidays, it appears, from the statement of those who superintended his health, that it suffered but little interruption until a few days previous to his departure for home.

The 2d of July, 1815, the day on which I revisited this patient, he was thus circumstanced:—His skin was hot and dry; pulse quick; appetite puny; restless, uneasy, and

---

unable to support the weight of his body beyond a few minutes in an erect posture; with some difficulty, he was prevailed on to walk the length of the room, that I might see how enfeebled his bodily actions had become; the remarkable lateral inclination of the body, and the feebleness of the lower extremities gave me a suspicion that the spinal column was in a diseased state, or about to become so. On a careful inspection of this part, that opinion could not be encouraged; there was, however, sufficient alteration in the conformation of the soft parts to indicate that considerable structural derangement had taken place, but of what nature or character, it was then difficult to determine.

It is here, Gentlemen, that I beg of you to pay some attention to the history of this singular and most extraordinary case.—Twelve months before this time, this child was a model of one of Nature's best works, well formed, active, and healthy, with a countenance indicative of future years—he had now suddenly lost his bloom, become dull, inactive, and fretful.

On placing him naked before me, for the purpose of seeing more clearly the state of the spine, the part which, as before observed, was suspected to be the seat of his disorder, a prominence below the spurious costæ on the left side shewed itself; reddened, and slightly sore to the touch: it appeared like a phlegmonous tumour in its incipient stage. There was no complaint made by the child of uneasiness in any other part; the belly was soft and flaccid: this enlargement excepted, all was externally right to the eye. A few days from this examination, he was removed from hence, and went under the care of an eminent physician, in a neighbouring county. Whilst under that gentleman's superintendance, his body quickly enlarged; and in consultation with a highly respectable and intelligent surgeon, the operation of paracentesis was resolved on, a distinct undulation of fluid being cognizable within the parietes of the abdomen. When this intention was announced to me by the father of the child, I was surprised at the necessity for the proposal of that operation, there having a week only before been not the slightest reason to apprehend the existence or approach of a deposition of fluid within the abdominal cavity. Two days after the receipt of this intelligence, I went, accompanied by Mr. ———, to see this patient whom, in the presence of the medical gentleman before mentioned, we attentively examined. At that time no fluctuation was discernible, neither was there any tumefaction in the left side, at the part where it had so visibly existed ten days before. But the whole abdomen was enormously enlarged, and the strength of the patient rapidly declining; the eyes were sunk; countenance pallid; hurried circulation: in short, death was evidently fast approaching, to seize upon this innocent victim.

On the day after his decease, which happened in about another week, permission was obtained to inspect the body. The following were the appearances on dissection.

When the cavity of the abdomen was exposed, as nearly as could be guessed, a quarter of a pint of a bloody fluid rushed out, accompanied by an highly intolerable fœtid gas.

The first and only viscus that came to view, was an enormously enlarged liver, which spread over the entire superficies of the abdomen, and occupied pretty nearly the whole of its cavity, as well as a large portion of that of the thorax, by pushing up the diaphragm apparently to its utmost stretch. There was only a small vacuum in the left inguen, which remained uncovered. The left lobe reached across the body completely, and formed, during life, that tumour which was perceptible beneath the false ribs on the left side.

Externally, this large mass of disease wore the appearance of an assemblage of small islands of different size, form, and colour; on dividing these with the knife, true pus flowed out in copious quantities. That portion of what had once been liver, which stretched upwards into the thorax, contained not less than four ounces of a puriform fluid: the remaining substance was a putrid disorganized mass, shapeless, and of varied hue. There was only an exceedingly small portion of this organ that retained its proper colour; and had it not been for this, and the attachment of the gall-bladder, (which yet retained healthy-looking bile) no anatomist could have recognized it as a human viscus.

Underneath the liver, a quart of bloody fluid, containing some large coagula, was found; it was this substance most likely that communicated, on percussion, the sensation of fluidity; and its retrocession behind the liver afterwards, most probably, was the reason why that sensation did not recur on repeating the trial a few days afterwards; change of posture might effect somewhat. The mesenteric glands were diseased, and in a suppurative state. The omentum was diseased also: it had the appearance of a slough about to be cast off from a gun-shot wound. The spleen was healthy, unaltered in size and colour, as were the rest of the abdominal contents.

Harwich, October 10, 1816.

